# Preferences for models of sexual health service delivery among gay, bisexual and other men who have sex with men in Australia: a discrete choice experiment

**DOI:** 10.1002/jia2.26482

**Published:** 2025-07-07

**Authors:** Jason J. Ong, Doug Fraser, Christopher Bourne, Andrew Grulich, Benjamin R. Bavinton

**Affiliations:** ^1^ School of Translational Medicine Monash University Melbourne Victoria Australia; ^2^ Melbourne Sexual Health Centre Alfred Health Melbourne Victoria Australia; ^3^ The Kirby Institute University of New South Wales Sydney New South Wales Australia

**Keywords:** general practice, HIV, LGBTQ, people living with HIV, sexually transmitted infections, testing

## Abstract

**Introduction:**

Gay, bisexual and other men who have sex with men are disproportionately affected by HIV and other sexually transmitted infections (STIs). This study explores preferences for different models of sexual health services among gay, bisexual and other men who have sex with men in Australia, using discrete choice experiments (DCEs).

**Methods:**

A cross‐sectional online survey was conducted from November 2022 to February 2023, targeting three groups: (1) gay, bisexual and other men who have sex with men living with HIV; (2) pre‐exposure prophylaxis (PrEP) users; and (3) non‐PrEP users. Participants were recruited through paid advertisements, sexual health clinics and community networks. The survey included demographic questions, sexual behaviour inquiries and three tailored DCEs to quantify preferences for service delivery attributes such as cost, type of clinic, appointment type, appointment frequency, extra services and where samples are taken for HIV/STI testing. We used latent class analyses to identify subgroups of people with similar preferences.

**Results:**

We recruited 1422 participants. The median age was 41 (interquartile range [IQR]: 32–54) for gay, bisexual and other men who have sex with men living with HIV (*N* = 396), 35 (IQR: 29–45) for PrEP users (*N* = 436) and 33 (IQR: 26–44) for non‐PrEP users (*N* = 590). In our latent class analyses, gay, bisexual and other men who have sex with men living with HIV preferred sexual health services to be delivered via sexual health clinics (46.2%), general practitioners (GP) with expertise in lesbian, gay, bisexual, trans, queer and others (LGBTQ+) health (33.0%) or were happy to go anywhere and to pay (20.7%). PrEP users preferred either PrEP‐only clinics or GP with expertise in LGBTQ+ health (75.2%) and GP with expertise in LGBTQ+ health only (24.8%). Non‐PrEP users preferred GP with expertise in LGBTQ+ health (44.7%) or any free service (22.8%); some did not want to test (22.2%) or were unsure of their preferences (10.2%).

**Conclusions:**

To align service models with client needs, investment in specialist sexual health clinics and LGBTQ+ competent GPs is important, though this may depend on local resources and infrastructure. Future research should focus on addressing financial barriers, evaluating telehealth and digital health interventions, and understanding testing reluctance among non‐PrEP users.

## 1 INTRODUCTION

Globally, gay, bisexual and other men who have sex with men (GBM) are disproportionately affected by HIV, accounting for a majority of new HIV diagnoses in high‐income countries despite making up a small percentage of the population [1]. In Australia, gay, bisexual and other men who have sex with men represent over 60% of cases of people living with HIV, highlighting the urgent need for tailored prevention and care services [2]. Evidence shows that tailored services may improve engagement in care and reduce HIV transmission [3, 4]. Thus, innovations in HIV care and prevention clinical services with a focus on differentiated service delivery in relation to individual needs and preferences could further contribute to reductions in the HIV burden of disease [5, 6]. Similarly, access to sexual health services is a powerful determinant of outcomes for sexually transmitted infections (STIs) [7].

To understand preferences for sexual health services among gay, bisexual and other men who have sex with men, there are three key groups: those living with HIV, pre‐exposure prophylaxis (PrEP) users and non‐PrEP users. Each group has distinct healthcare needs and service utilisation patterns. Gay, bisexual and other men who have sex with men living with HIV require regular HIV care, including viral load monitoring, antiretroviral therapy adherence support and routine STI screenings [8]. PrEP users engage with healthcare primarily for ongoing PrEP prescriptions, quarterly HIV/STI screenings and monitoring for medication side effects. Non‐PrEP users are recommended to have at least annual HIV/STI screening or more frequent testing if they have multiple partners [9]. These distinct healthcare needs underscore the importance of differentiated and tailored service delivery models.

Sexual health services are traditionally delivered through specialised sexual health clinics that are predominantly located in major cities. Alternatively, any general practitioner can also offer sexual health services. While general practitioners (GPs) are widely accessible, the fear of stigma and homophobia may prevent individuals from seeking care [10, 11]. Individuals can access limited sexual health services (e.g. HIV/STI screening) through online services or community‐based organisations [12]. During the coronavirus disease 19 (COVID‐19) pandemic, sexual health services adapted by expanding telehealth and digital services to enhance accessibility [13, 14, 15, 16]. While these non‐face‐to‐face models were necessary during restrictions, it remains unclear whether they align with the long‐term preferences of GBM, who may now favour a return to in‐person care for STI testing and provider interaction. Thus, it is timely to examine such preferences given the ways in which the COVID‐19 pandemic necessitated the rapid implementation of novel models of care [16].

## 2 METHODS

### 2.1 Study design and participants

Discrete choice experiments (DCEs) can quantify preferences between choices with multiple components [17]. By systematically assessing preferences for different aspects of service delivery, like appointment type, frequency and location of sample collection, DCEs can provide insights into client‐centred service delivery models. The DCE was used to assess trade‐offs rather than absolute preferences. For example, while few people would “prefer” to pay $150 versus receiving a service for free, the analysis helps quantify price sensitivity, that is how changes in cost impact service utilisation.

We conducted a cross‐sectional online survey of attitudes to sexual healthcare delivery using Qualtrics (Provo, UT, USA) from November 2022 to February 2023. Participants were eligible if they: were living in Australia; self‐identified as gay, bisexual and other men who have sex with men (inclusive of transgender men); were at least 18 years of age; and had a clinical appointment, including telehealth, for HIV management or sexual health screening in Australia since March 2020. The survey took approximately 10 minutes to complete and participants were offered the chance to opt‐in for a prize draw for one of ten $200 vouchers after submitting the survey. The survey was promoted using paid advertising on Grindr (a gay dating app), by direct contact to patients of sexual health clinics in New South Wales and Victoria, and unpaid promotions through networks of people living with HIV and gay, bisexual and other men who have sex with men. Before starting the survey, participants provided consent by confirming the statement “I agree to participate and have read the Participant Information Statement, begin survey.”

### 2.2 Measures

All participants provided demographic information (age, education, occupation, income, country of birth, sexuality). They were also asked about their relationship status, sexual behaviour (number of sexual partners, condom use, group sex). Participants were asked about their HIV status and PrEP use, and were organised into three different groups based on their responses: gay, bisexual and other men who have sex with men living with HIV, PrEP users and non‐PrEP users. Based on which group participants belonged, they were asked questions about their experience of and attitudes to sexual healthcare delivery and preferences for the future. Participants were asked where they usually went for HIV care/sexual healthcare/PrEP and were asked a series of questions about their attitudes to different elements of healthcare delivery as well as about their satisfaction with different modalities of consultation (face‐to‐face, telephone, video call) on a scale of 1 (very unsatisfied) to 5 (very satisfied).

### 2.3 Discrete choice experiment

**Figure 1:**
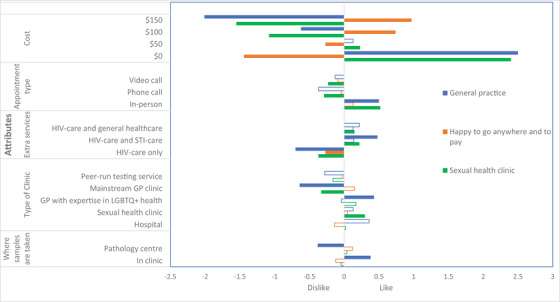
**Latent class analysis of preferences of sexual health service delivery from gay, bisexual and other men who have sex with men living with HIV**. *Notes*: Colour‐filled bars = level is statistically significant (*p*‐value < 0.05). No‐filled bars = level is not statistically significant (*p*‐value ≥ 0.05). Currency is in Australian dollars (1 AUD = 0.64 USD). GBM, gay, bisexual and other men who have sex with men; GP, general practitioner; LGBTQ+, lesbian, gay, bisexual, trans, queer and others; PrEP, pre‐exposure prophylaxis; STI, sexually transmitted infection.

The survey included three DCEs to quantify preferences for service delivery: one for gay, bisexual and other men who have sex with men living with HIV, one for PrEP users and one for non‐PrEP users. A literature review was performed to identify potential attributes related to accessing HIV care and prevention and sexual health services for gay, bisexual and other men who have sex with men. We conducted a structured consultation of the potential attributes with a group of experts including researchers, clinicians and community representatives who ranked the attributes according to their importance and were given the opportunity to suggest additional attributes and amendments. The final list of attributes and levels is presented in Table S1. The survey was pilot‐tested with five in‐house staff identifying as gay men to assess usability, clarity and completion time, leading to only minor wording refinements in the choice set introductions without major modifications. Each participant was presented with six choice sets (Figure 1). Within each choice set respondents were asked to choose their preferred option from two options and if they did not like either options, they can choose to opt‐out. Gay, bisexual and other men who have sex with men living with HIV were asked “Thinking about your routine clinical appointments for HIV care in the future, which option do you prefer?” PrEP users were asked “Thinking about your routine clinical appointments for PrEP in the future, which option do you prefer?”. Non‐PrEP users were asked “Thinking about your routine clinical appointments for asymptomatic sexual health check‐ups in the future, which option do you prefer?”

### 2.4 Statistical analysis

We used descriptive statistics to summarise socio‐demographic characteristics. We analysed the choice data using latent class conditional logit regression models to explore similarities (or “classes”) in preference behaviour. The number of classes was determined based on the Akaike Information Criteria and interpretability. We hypothesised *a priori* that the following characteristics may influence preferences: education level, country of birth, work status, number of sexual partners, age and consistency of condom use. Statistical significance was defined as *p*‐value < 0.05. We calculated the relative importance of each attribute using the coefficient range of an attribute divided by the sum of ranges from all attributes [18]. The relative importance indicates how much weight an attribute has in the decision to use the HIV/sexual health service. We used NLOGIT (version 6, Econometric Software Inc, USA) for the model estimations.

### 2.5 Ethics approval

Ethical approval was obtained from the University of New South Wales (UNSW) Sydney Human Research Ethics Committee (HC210939). We present our study according to the STROBE statement.

## 3 RESULTS

Of the 1657 participants who consented to participate and met eligibility requirements, 1422 participants (85.8%) completed at least one DCE choice set and were included in this analysis. Most participants (72.6%) were recruited through paid advertising (Grindr), while the remaining participants were recruited through publicly funded sexual health services (19.3%) and unpaid promotions (8.1%). Unpaid promotions included anything that was not a paid ad, for example the clinics sending out emails/messages.

### 3.1 Study population characteristics

Table 1 summarises the characteristics of the participants. The median age for gay, bisexual and other men who have sex with men living with HIV was 41 (interquartile range [IQR]: 32–54), PrEP users was 35 (IQR: 29–45) and non‐PrEP users was 33 (IQR: 26–44). The median number of years living with HIV was 8 (IQR: 4–17). Most identified as gay (people with HIV = 83.6%; PrEP users = 80.5%; people without HIV and not on PrEP = 57.1%). The majority usually attended a sexual health clinic (gay, bisexual and other men who have sex with men living with HIV = 73.5%; PrEP users = 58.3%) or a general practitioner (non‐PrEP users = 57.1%). The majority were born in Australia (gay, bisexual and other men who have sex with men living with HIV = 54.0%; PrEP users = 50.2%; and non‐PrEP users = 51.7%); while a substantial proportion were born overseas (those living with HIV: 32.8%, PrEP users: 31.7%, non‐PrEP users: 24.7%).

**Table 1:** Study participant socio‐demographic characteristics and sexual behaviours, comparing gay, bisexual and other men who have sex with men living with HIV, PrEP users and non‐PrEP users

Characteristic	GBM living with HIV *n* = 396 *n* (%)^a^	PrEP users (*n* = 436) *n* (%)^a^	Non‐PrEP users (*n* = 590) *n* (%)^a^
**Usual services attended** ^b^			
General Practice clinic	88 (22.2)	219 (50.2)	337 (57.1)
Sexual health clinic	291 (73.5)	254 (58.3)	230 (39.0)
Hospital	50 (12.6)	16 (3.7)	49 (8.3)
Other	6 (1.5)	25 (5.7)	29 (4.9)
Missing data^c^	26	23	61
**Country of birth**			
Australia	214 (54.0)	219 (50.2)	305 (51.7)
Overseas	130 (32.8)	138 (31.7)	146 (24.7)
Missing data	52 (13.1)	79 (18.1)	139 (23.6)
**Gay postcode categories** ^d^			
> 10% gay	26 (6.6)	54 (12.4)	28 (4.7)
5–9.9% gay	82 (20.7)	64 (14.7)	57 (9.7)
< 5%	231 (58.3)	240 (55.0)	371 (62.9)
Missing data	57 (14.4)	78 (17.9)	134 (22.7)
**Highest education level**			
Up to year 10	30 (7.6)	23 (5.3)	67 (11.4)
Up to year 12	54 (13.6)	51 (11.7)	88 (14.9)
Tertiary diploma or trade certificate	106 (26.8)	76 (17.4)	127 (21.5)
University degree	167 (42.2)	216 (49.5)	187 (31.7)
Missing data	39 (9.8)	70 (16.1)	121 (20.5)
**Current occupation**			
Employed full time	215 (54.3)	245 (56.2)	224 (38.0)
Employed part‐time	41 (10.4)	41 (9.4)	89 (15.1)
Pension/social security	34 (8.6)	14 (3.2)	38 (6.4)
Student	15 (3.8)	38 (8.7)	38 (6.4)
Unemployed	24 (6.1)	13 (3.0)	54 (9.2)
Missing data	67 (16.9)	85 (19.5)	147 (24.9)
**Annual personal income**			
< $20,000	68 (17.2)	48 (11.0)	132 (22.4)
$20,000−$49,999	93 (23.5)	65 (14.9)	122 (20.7)
$50,000−$99,999	133 (33.6)	155 (35.6)	150 (25.4)
≥ $100,000	62 (15.7)	97 (22.2)	66 (11.2)
Missing data	40 (10.1)	71 (16.3)	120 (20.3)
**Number of male sexual partners in the last 6 months**			
None	53 (13.4)	12 (2.8)	58 (9.8)
1	71 (17.9)	14 (3.2)	54 (9.2)
2−10	158 (39.9)	193 (44.3)	271 (45.9)
> 10	65 (16.4)	135 (31.0)	54 (9.2)
Missing data	49 (12.4)	82 (18.8)	153 (25.9)
**Condom use with casual partners**			
Never	114 (28.8)	125 (28.7)	101 (17.1)
Occasionally	79 (19.9)	148 (33.9)	114 (19.3)
Often	38 (9.6)	44 (10.1)	66 (11.2)
Always	38 (9.6)	16 (3.7)	94 (15.9)
No sex in the past 6 months	47 (11.9)	8 (1.8)	51 (8.6)
No casual partners	31 (7.8)	6 (1.4)	19 (3.2)
Missing data	41(10.4)	74 (17.0)	127 (21.5)

Abbreviations: GBM, gay, bisexual and other men who have sex with men; PrEP, pre‐exposure prophylaxis for HIV.

^a^ Percentages use total population as the denominator. Thus, the percentage of missing data can be calculated if needed.

^b^ Participants can choose more than one option for this question.

^c^ Missing data here are respondents who did not respond to at least one of these options.

^d^ Postcodes of residence were categorised based on the estimated resident gay male population [19].

### 3.2 Satisfaction with sexual health services

Table 2 summarises participants’ satisfaction with modalities of receiving sexual healthcare. In general, individuals were more satisfied with face‐to‐face interactions (range: 88.1−92.5%) than telephone (range: 74.8−86%) or video calls (range: 63.8−82.4%). Chi‐square tests comparing face‐to‐face consultations with telephone or video calls across the three groups showed statistically significant differences for each of the comparisons (*p*‐values < 0.05). Regarding STI testing, individuals were more satisfied with public sexual health clinics (range: 90.5−94.2%) and general practice clinics with expertise in lesbian, gay, bisexual, trans, queer and others (LGBTQ+) health (range: 89.3−99.2%), and had the lowest satisfaction with mainstream general practice clinics (range: 79.1−82.3%).

**Table 2:** Satisfaction^a^ with current HIV and sexual healthcare, comparing gay, bisexual and other men who have sex with men living with HIV, PrEP users and non‐PrEP users

Characteristic	GBM living with HIV	PrEP users	Non‐PrEP users
	***n* (%)** ^b^	***n* (%)** ^b^	***n* (%)** ^b^
**Modality**			
Face‐to‐face	318 (88.1)	322 (92.5)	363 (89.2)
Telephone	235 (74.8)	202 (86.0)	238 (82.9)
Video call	127 (63.8)	108 (82.4)	148 (77.1)
**STI test from the following services**			
Public sexual health clinic	306 (94.2)	277 (93.0)	316 (90.5)
General practice clinic with expertise in LGBTQ+ health	234 (99.2)	223 (95.3)	266 (89.3)
Mainstream general practice clinic	174 (79.1)	196 (77.8)	274 (82.3)
Peer‐run community‐based testing	141 (84.9)	135 (90.0)	202 (88.6)
Pathology collection centre	171 (82.2)	189 (87.9)	241 (88.9)
Own home	101 (77.1)	86 (83.5)	162 (86.6)

Abbreviations: GBM, gay, bisexual and other men who have sex with men; LGBTQ+, lesbian, gay, bisexual, trans, queer and others; PrEP, pre‐exposure prophylaxis; STI, sexually transmitted infection.

^a^ Satisfaction was assessed using a 5‐point Likert scale. We collapsed the categories of “very satisfied” and “satisfied” as the outcome of this table.

^b^ Denominator removes those with no experience in this mode.

### 3.3 Preferences of gay, bisexual and other men who have sex with men living with HIV

Figure 1 (and Table S2) demonstrates three latent classes: “Sexual Health Clinic,” “General Practice” and “Happy to go anywhere and to pay.” For those in the “Sexual Health Clinic” class (46.2%), they preferred attending a sexual health clinic that is low‐cost (up to $50) or free, in‐person, and includes HIV, STI and general healthcare. For those in the “General Practice” class (33.0%), they preferred their GP to have expertise in LGBTQ+ health and for services to be free, in‐person, including HIV and STI care, and for staff in clinic to take the samples for STI testing (rather than going to a pathology centre). For those in the “Happy to go anywhere and to pay” class (20.7%), they were willing to pay up to $150 and disliked receiving only HIV care. Participants in this group were more likely to be aged < 25 years old, report inconsistent condom use and be born in Australia. Figure S1 demonstrates that cost considerations had the highest relative importance: “Sexual Health Clinic” class (67%), “Happy to go anywhere and to pay” class (68%) and “General Practice” class (54%).

### 3.4 Preferences of PrEP users

**Figure 2:**
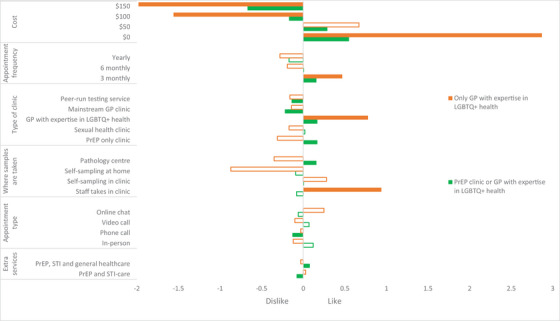
**Latent class analysis of preferences of sexual health service delivery from PrEP users**. *Notes*: Colour‐filled bars = level is statistically significant (*p*‐value < 0.05). No filled bars = level is not statistically significant (*p*‐value ≥ 0.05). Currency is in Australian dollars (1 AUD = 0.64 USD). GP, general practitioner; LGBTQ+, lesbian, gay, bisexual, trans, queer and others; PrEP, pre‐exposure prophylaxis; STI, sexually transmitted infection.

Figure 2 (and Table S3) demonstrates two latent classes: “PrEP clinic or GP with expertise in LGBTQ+ health” and “Only GP with expertise in LGBTQ+ health.” For those in the “PrEP clinic or GP with expertise in LGBTQ+ health” class (75.2%), they preferred services to be low‐cost (up to $50) or free, 3‐monthly, provide samples for STI testing at a pathology centre and disliked phone‐based consultations. For those in the “Only GP with expertise in LGBTQ+ health” class (24.8%), they were more price‐sensitive than those in the first class (i.e. had 5.2 times greater preference for a free service), preferred 3‐monthly visits and preferred for staff in clinic to take the samples for STI testing. Figure S2 demonstrates that cost considerations had the highest relative importance: “PrEP clinic or GP with expertise in LGBTQ+ health” class (47%) and “Only GP with expertise in LGBTQ+ health” class (54%).

### 3.5 Preferences of non‐PrEP users

**Figure 3:**
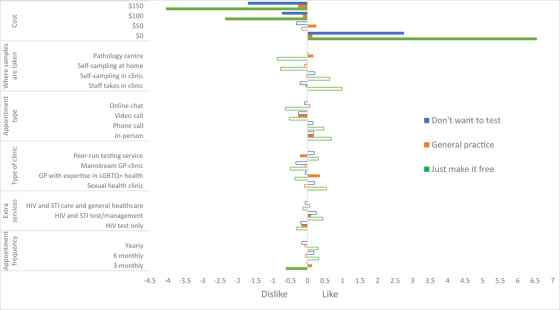
**Latent class analyses of preferences for sexual health service delivery from non‐PrEP users**. *Notes*: Colour‐filled bars = level is statistically significant (*p*‐value < 0.05). No‐filled bars = level is not statistically significant (*p*‐value ≥ 0.05). Currency is in Australian dollars (1 AUD = 0.64 USD). Data from class 4 is not shown, given the wide range of statistically insignificant coefficients (for details, see Table S4). GP, general practitioner; LGBTQ+, lesbian, gay, bisexual, trans, queer and others; PrEP, pre‐exposure prophylaxis; STI, sexually transmitted infection.

Figure 3 (and Table S4) demonstrates four latent classes: “Just make it free,” “General Practice,” “Don't want to test” and “Unsure about preferences.” For those in the “Just make it free” class (22.8%), they preferred free sexual health services, no matter what form this took. For those in the “General practice” class (44.7%), they preferred their GP to have expertise in LGBTQ+ health, were willing to pay $50 for the service (i.e. had 1.8 times greater preference to pay $50 than for a free service), wanted to attend clinics in‐person, preferred 3‐monthly visits and preferred for visits to include both HIV and STI testing/management. For those in the “Don't want to test” class (22.2%), they were more likely to choose opt‐out (i.e. not attend any service) and preferred free services. For those in the “Unsure about preferences” class (10.2%), there was a preference for self‐sampling in the clinic and provision of both HIV and STI testing/management, but other attributes had wide standard errors. Figure S3 demonstrates that cost considerations had the highest relative importance: “Just make it free” class (64%), “General practice” class (23%) and “Don't want to test” class (67%). For the “unsure about preferences” class, the highest relative importance was for appointment type (43%).

## 4 DISCUSSION

This study provides valuable insights into the preferences for sexual health service delivery among gay, bisexual and other men who have sex with men in Australia, revealing the distinct needs of individuals based on their HIV status and use of PrEP. Among gay, bisexual and other men who have sex with men living with HIV, the most preferred service models included sexual health clinics (46.2%), followed by GPs with LGBTQ+ expertise (33.0%), while a smaller group (20.7%) expressed flexibility to access services anywhere, even with out‐of‐pocket costs. PrEP users showed a strong preference for either PrEP‐only clinics or LGBTQ+ competent GPs (75.2%), with 24.8% preferring LGBTQ+ competent GPs exclusively. For non‐PrEP users, preferences were more varied: 44.7% favoured LGBTQ+ competent GPs, 22.8% prioritised free services, while 22.2% were reluctant to test, and 10.2% were unsure about their preferences. Our findings add to the literature by underscoring the necessity for health service models to align with client expectations, emphasising accessibility, affordability and provider expertise in LGBTQ+ health. While these preference data provide valuable insights into service acceptability and engagement, they should be considered alongside other critical factors, including clinical effectiveness, feasibility, cost and healthcare system constraints.

Our findings highlight the diverse therapeutic itineraries undertaken by gay, bisexual and other men who have sex with men in Australia when seeking sexual health services. The concept of therapeutic itineraries—which refers to the dynamic and individualised pathways individuals navigate through healthcare systems—offers a useful lens to understand how different service models align with client needs and expectations. The latent class analysis in our study underscores that service preferences are shaped not only by accessibility and affordability but also by perceptions of provider expertise, stigma and prior healthcare experiences. While sexual health clinics remain a preferred option, particularly for gay, bisexual and other men who have sex with men living with HIV, our results also indicate a strong preference for GPs with LGBTQ+ expertise, reflecting the importance of trust, continuity of care and holistic health management. There were also notable socio‐demographic differences between the three study populations, which may have influenced their healthcare preferences. For example, those living with HIV tended to be older, had higher proportions of full‐time employment and were more likely to attend sexual health clinics, while PrEP users were generally younger, more educated and more likely to use LGBTQ+ competent GPs. In contrast, non‐PrEP users showed more variation in income, education and care‐seeking patterns, with a substantial proportion unsure of their preferences or reluctant to test. These differences highlight the importance of considering socio‐demographic context when interpreting service delivery preferences, as structural and social factors likely shape healthcare access and engagement. These findings resonate with broader discussions in healthcare service research that emphasise differentiated service delivery models as key to improving health outcomes and engagement in care. Future research should further explore how structural and psychosocial factors influence the therapeutic itineraries of diverse subgroups within the community, particularly those who remain hesitant or disengaged from routine sexual health screening.

Cost emerged as a critical factor influencing the choice of sexual health services, which aligns with global evidence on the importance of minimising out‐of‐pocket expenses to increase the uptake of sexual health services [20]. In Australia, HIV treatment is subsidised under the Pharmaceutical Benefits Scheme for anyone living in Australia, regardless of their visa status. However, out‐of‐pocket costs may still apply for consultations depending on whether a person accesses care through general practice or sexual health clinics. HIV and STI screening is free at publicly funded sexual health clinics but may incur fees in general practice or private clinics. These cost variations could partly explain differences in service preferences across the three groups. For instance, people living with HIV, who require ongoing specialist HIV care and routine monitoring, were more likely to prioritise access to specialised sexual health clinics, which typically provide free or low‐cost services. PrEP users, who need regular 3‐monthly HIV/STI testing, showed a strong preference for low‐cost PrEP‐only clinics or LGBTQ+ competent GPs, as cost and accessibility directly impact their adherence. In contrast, non‐PrEP users had a wider range of preferences, with some prioritising free services, while others were less engaged in regular testing, possibly due to financial barriers or lower perceived risk. Overall, there is strong evidence that providing free or low‐cost services for the public control of infectious diseases provides significant returns on investment [21]. Testing and managing infectious diseases earlier reduces morbidity and prevents ongoing transmission, particularly reaching those who are disproportionally affected, who are often marginalised populations with lower socio‐economic status. For example, the estimated lifetime cost of managing a single case of HIV in Australia is approximately $282,000, underscoring the potential economic return of investing in prevention and equitable service delivery [22]. In designing sexual health services, we must consider the influence of out‐of‐pocket costs impacting the accessibility of these services, particularly for those who would benefit most, who are often socio‐economically disadvantaged [6].

Preferences for service delivery location varied significantly across subgroups. While many participants favoured specialised sexual health clinics, a notable proportion of gay, bisexual and other men who have sex with men indicated a preference for accessing care through GPs with expertise in LGBTQ+ health. LGBTQ+ competent GPs are more likely to provide inclusive and affirming care, including gender‐appropriate language, non‐judgemental communication and familiarity with LGBTQ+ health concerns. Stigma in mainstream healthcare settings remains a barrier, with some reporting discomfort in disclosing their sexual history, HIV status or PrEP use due to fear of judgement or discrimination [23, 24]. This preference underscores the pivotal role that GPs can play in expanding access to sexual health services, especially in areas where specialised clinics may not be available. We acknowledge that the availability and accessibility of services are strongly influenced by geographical location, and even some urban centres may experience limited access to sexual health clinics. Mapping service availability across different regions remains complex, and this issue is currently being addressed through ongoing workforce planning initiatives led by the College of Physicians to improve equitable service distribution. This further reinforces the importance of equipping a broader range of GPs with LGBTQ+ health expertise to address geographic disparities in care. Most Australians see a GP at least once a year (82% in 2023) [25]. However, current limitations in GP training on HIV and sexual healthcare present challenges that need to be addressed through targeted educational interventions [26] and support models [27] to ensure high‐quality care. Our study also sheds light on the evolving preferences shaped by the COVID‐19 pandemic, which accelerated the adoption of telehealth and digital health services. While some participants expressed satisfaction with telehealth, face‐to‐face consultations remained the preferred mode of service delivery. While telehealth can enhance access to care, its role should be assessed in the context of client needs, clinical effectiveness and service feasibility.

Our study's strengths include a large and diverse sample and the use of DCEs to quantitatively assess preferences, providing robust insights into service delivery attributes that matter most to gay, bisexual and other men who have sex with men. However, limitations should be considered when interpreting these findings. First, our recruitment was primarily from Grindr and publicly funded sexual health services so we may not have adequately captured preferences from participants who are less likely to use these apps and services. The reliance on Grindr‐based recruitment (72.6%) may overrepresent tech‐savvy, sexually active men while underrepresenting those less digitally connected. Future research should explore alternate recruitment strategies, such as community‐based sampling or non‐digital outreach, to capture a more diverse representation. Second, we used self‐reported data which may be prone to social desirability and recall bias, inherent in self‐reported surveys. To mitigate this, the surveys were voluntary and anonymous, recall time frames were relatively short and we pilot‐tested the survey to ensure questions were framed appropriately. Future research should explore the reasons behind specific service preferences through qualitative methods, which would further enhance our understanding of the drivers of service uptake. Third, our study is conducted in the socio‐economic context of Australia and may not be generalisable to other countries. Similarly, preferences of populations (e.g. heterosexual populations) not included in our study populations may differ. Thus, conducting health preference research in other countries and sub‐populations would be valuable in identifying their preferences for sexual health service delivery. Fourth, our study was only available in English, and although almost half were born outside of Australia, we have not captured preferences from those not fluent in English. Future studies could target non‐English speakers living in Australia. Finally, we did not collect data on participants’ current accessibility to services (e.g. rural vs. urban), which limits our ability to assess how service accessibility may influence preferences. Future research should explore how geographic access shapes service utilisation and preferences.

## 5 CONCLUSIONS

Overall, this research highlights the need for tailored sexual health service models that address financial barriers and leverage GPs’ potential to provide inclusive and competent care for gay, bisexual and other men who have sex with men. Addressing these preferences will be crucial in ensuring equitable access to HIV and sexual health services, ultimately contributing to better health outcomes in the community.

## COMPETING INTERESTS

JJO and BRB have received honoraria and travel support from Gilead, unrelated to this project. All other authors declare no conflicts of interests.

## AUTHORS’ CONTRIBUTIONS

BRB and JJO conceived and designed the study. DF and JJO contributed to the data collection. JJO analysed the data and drafted the manuscript. All authors contributed to the interpretation of the data, provided critical revisions to the manuscript and approved the final version of the manuscript.

## FUNDING

This research was funded by a grant from Gilead Sciences, Inc. (RG203102). The funder had no role in the conception, design, data collection, analysis or manuscript.

## Supporting information




**Table S1**: Final attributes and levels in the DCE.
**Table S2**: Latent class analysis of preferences for sexual health services for people living with HIV.
**Table S3**: Latent class analysis of preferences of PrEP users.
**Table S4**: Latent class analysis of preferences of people without HIV and not using PrEP.
**Figure S1**: Relative importance of preferences for people living with HIV.
**Figure S2**: Relative importance of preferences for PrEP users.
**Figure S3**: Relative importance of preferences of GBM without HIV and not using PrEP.

## Data Availability

The data that support the findings of this study are available from the corresponding author upon reasonable request.
