# Supplementary material for: Preferences for models of sexual health service delivery among gay, bisexual and other men who have sex with men in Australia: a discrete choice experiment
Source: J Int AIDS Soc. 2025 Jul 7;28(Suppl 3):e26482. doi: 10.1002/jia2.26482 (PMC12232476; doi:10.1002/jia2.26482)
Supplement: Supplementary file 1 — Table S1: Final attributes and levels in the DCE. Table S2: Latent class analysis of preferences for sexual health services for people living with HIV. Table S3: Latent class analysis of preferences of PrEP users. Table S4: Latent class analysis of preferences of people without HIV and not using PrEP. Figure S1: Relative importance of preferences for people living with HIV. Figure S2: Relative importance of preferences for PrEP users. Figure S3: Relative importance of preferences of GBM without HIV and not using PrEP. [file JIA2-28-e26482-s001.docx]

Table of Contents

[Supplementary Table S1 Final attributes and levels in the DCE 2](#_Toc195775289)

[Supplementary Table S2 Latent class analysis of preferences for sexual health services for people living with HIV 4](#_Toc195775290)

[Supplementary Table S3 Latent class analysis of preferences of PrEP users 5](#_Toc195775291)

[Supplementary Table S4 Latent class analysis of preferences of people without HIV and not using PrEP 6](#_Toc195775292)

[Supplementary Figure S1 Relative importance of preferences for people living with HIV 7](#_Toc195775293)

[Supplementary Figures S2 Relative importance of preferences for PrEP users 8](#_Toc195775294)

[Supplementary Figure S3 Relative importance of preferences of GBM without HIV and not using PrEP 9](#_Toc195775296)

# Supplementary Table S1 Final attributes and levels in the DCE

| **GBM living with HIV** | |
| --- | --- |
| **Attributes** | **Levels** |
| *What type of clinic* | HIV specialist clinic in a hospital  Public sexual health clinic  GP clinic with expertise in LGBTQ+ health  Mainstream GP clinic  Peer-run testing service (e.g. a[TEST], PRONTO!) |
| *Where is the appointment* | In-person at the clinic  Phone call  Video call |
| *What services are included in each appointment* | HIV care only  HIV care and STI testing and treatment  HIV care and general healthcare |
| *Where are the blood, urine, and swab samples taken for HIV and STI tests* | In the clinic  At a pathology collection centre (eg Laverty, Dorevitch, QML) |
| *Total out-of-pocket cost* | $0  $50  $100  $150 |
| **HIV-negative GBM on PrEP** |  |
| *What type of clinic* | PrEP-only clinic  Public sexual health clinic  GP clinic with expertise in LGBTQ+ health  Mainstream GP clinic  Peer-run testing service (e.g. a[TEST], PRONTO!) |
| *Where is the appointment* | In-person at the clinic  Phone call  Video call  Online chat |
| *How often are the appointments* | Every 3 months  Every 6 months  Once per year |
| *What services are included in each appointment* | HIV/STI testing and PrEP  HIV/STI testing, PrEP, and general healthcare |
| *Where are the blood, urine, and swab samples taken for HIV and STI tests* | Staff take samples in the clinic  Take my own samples in the clinic  Take my own samples at home  At a pathology collection centre (eg Laverty, Dorevitch, QML) |
| *Total out-of-pocket cost* | $0  $50  $100  $150 |
| **HIV-negative/untested GBM not on PrEP** |  |
| *What type of clinic* | Public sexual health clinic  GP clinic with expertise in LGBTQ+ health  Mainstream GP clinic  Peer-run testing service (e.g. a[TEST], PRONTO!) |
| *Where is the appointment* | In-person at the clinic  Phone call  Video call  Online chat |
| *How often are the appointments* | Every 3 months  Every 6 months  Once per year |
| *What services are included in each appointment* | HIV testing only  HIV testing and STI screening/treatment  HIV testing, STI screening/treatment, and general healthcare |
| *Where are the blood, urine, and swab samples taken for HIV and STI tests* | Staff take samples in the clinic  Take my own samples in the clinic  Take my own samples at home  At a pathology collection centre (eg Laverty, Dorevitch, QML) |
| *Total out-of-pocket cost* | $0  $50  $100  $150 |

GBM = gay, bisexual and other men who have sex with men; GP = general practice; LGBTQ+ = lesbian, gay, bisexual, trans, queer and others; PrEP = pre-exposure prophylaxis; STI = sexually transmitted infections

# Supplementary Table S2 Latent class analysis of preferences for sexual health services for people living with HIV

|  | “Sexual Health Clinic”  (46.2%) | | | “Not fussed where to go and happy to pay”  (20.7%) | | | “General Practice”  (33.0%) | | |
| --- | --- | --- | --- | --- | --- | --- | --- | --- | --- |
|  | Coefficient |  | SE | Coefficient |  | SE | Coefficient |  | SE |
| **Cost (AUD)** |  |  |  |  |  |  |  |  |  |
| $0 | 2.40 | *** | 0.12 | -1.44 | *** | 0.25 | 2.50 | *** | 0.22 |
| $50 | 0.23 | *** | 0.08 | -0.27 | ** | 0.12 | 0.13 |  | 0.24 |
| $100 | -1.08 | ** | 0.09 | 0.74 | *** | 0.15 | -0.62 | ** | 0.27 |
| $150 | -1.55 | *** | 0.12 | 0.97 | *** | 0.19 | -2.01 | *** | 0.49 |
| **Appointment type** |  |  |  |  |  |  |  |  |  |
| In-person | 0.52 | *** | 0.08 | 0.13 |  | 0.13 | 0.50 | *** | 0.17 |
| Phone call | -0.29 | *** | 0.29 | -0.04 |  | 0.14 | -0.37 | * | 0.19 |
| Video call | -0.23 | *** | 0.08 | -0.09 |  | 0.13 | -0.13 |  | 0.19 |
| **Extra services** |  |  |  |  |  |  |  |  |  |
| HIV-care only | -0.37 | *** | 0.07 | -0.27 | ** | 0.12 | -0.70 | *** | 0.18 |
| HIV-care and STI-care | 0.22 | *** | 0.07 | 0.14 |  | 0.11 | 0.48 | *** | 0.15 |
| HIV-care and general healthcare | 0.15 | ** | 0.07 | 0.13 |  | 0.11 | 0.22 |  | 0.13 |
| **Type of clinic** |  |  |  |  |  |  |  |  |  |
| Hospital | 0.02 |  | 0.09 | -0.14 |  | 0.17 | 0.36 | * | 0.19 |
| Sexual health clinic | 0.30 | ** | 0.12 | 0.05 |  | 0.18 | 0.13 |  | 0.19 |
| GP with expertise in LGBTQ+ health | 0.17 | * | 0.10 | -0.04 |  | 0.19 | 0.43 | ** | 0.18 |
| Mainstream GP clinic | -0.33 | *** | 0.10 | 0.15 |  | 0.18 | -0.64 | *** | 0.21 |
| Peer-run testing service | -0.16 |  | 0.11 | -0.02 |  | 0.16 | -0.28 |  | 0.23 |
| **Where samples are taken** |  |  |  |  |  |  |  |  |  |
| In clinic | -0.04 |  | 0.07 | -0.12 |  | 0.12 | 0.38 | *** | 0.12 |
| Pathology centre | 0.04 |  | 0.07 | 0.12 |  | 0.12 | -0.38 | *** | 0.12 |
| **Opt out** | -1.55 | *** | 0.16 | -3.59 | *** | 0.18 | 2.50 | *** | 0.24 |
| **Theta in class probability model** |  |  |  |  |  |  |  |  |  |
| Age <25 | 0.45 |  | 0.29 | 1.21 | *** | 0.22 | - |  |  |
| Inconsistent condom use | 0.18 |  | 0.19 | 0.89 | ** | 0.39 | - |  |  |
| Born in Australia | 0.44 | * | 0.26 | 0.53 | ** | 0.2 | - |  |  |
| Log-likelihood function | -1802 |  |  |  |  |  |  |  |  |
| AIC/N | 1.581 |  |  |  |  |  |  |  |  |

* p value <0·10, ** p value <0·05, *** p value <0·01; ^#^ The coefficient for the reference group is calculated as the negative sum of the other coefficients.

Currency is in Australian dollars (1 AUD=0.64 USD).

AIC = Akaike information criteria; GP = General practitioner; HIV = Human immunodeficiency virus; LGBTQ+ = lesbian, gay, bisexual, trans and queer/questioning, and more identities; PrEP = pre-exposure prophylaxis (for HIV); SE = standard error; STI = sexually transmitted infections

# Supplementary Table S3 Latent class analysis of preferences of PrEP users

|  | “PrEP clinic or  GP with expertise in LGBTQ+ health”  (75.2%) | | | “Only GP with expertise in  LGBTQ+ health”  (24.8%) | | |
| --- | --- | --- | --- | --- | --- | --- |
|  | Coefficient |  | SE | Coefficient |  | SE |
| **Cost (AUD)** |  |  |  |  |  |  |
| $0 | 0.55 | *** | 0.06 | 2.87 | *** | 0.19 |
| $50 | 0.29 | *** | 0.05 | 0.67 |  | 0.51 |
| $100 | -0.17 | *** | 0.05 | -1.56 | ** | 0.65 |
| $150 | -0.67 | *** | 0.06 | -1.98 | *** | 0.74 |
| **Appointment frequency** |  |  |  |  |  |  |
| 3 monthly | 0.16 | *** | 0.04 | 0.47 | *** | 0.13 |
| 6 monthly | 0.01 |  | 0.04 | -0.19 |  | 0.26 |
| Yearly | -0.17 | *** | 0.04 | -0.28 |  | 0.20 |
| **Type of clinic** |  |  |  |  |  |  |
| PrEP only clinic | 0.17 | ** | 0.08 | -0.31 |  | 0.25 |
| Sexual health clinic | 0.02 |  | 0.06 | -0.17 |  | 0.26 |
| GP with expertise in LGBTQ+ health | 0.17 | *** | 0.06 | 0.78 | *** | 0.27 |
| Mainstream GP clinic | -0.22 | *** | 0.06 | -0.14 |  | 0.27 |
| Peer-run testing service | -0.14 | * | 0.08 | -0.16 |  | 0.30 |
| **Where samples are taken** |  |  |  |  |  |  |
| Staff takes in clinic | -0.08 |  | 0.09 | 0.94 | *** | 0.24 |
| Self-sampling in clinic | 0.01 |  | 0.09 | 0.28 |  | 0.41 |
| Self-sampling at home | -0.09 |  | 0.08 | -0.87 | * | 0.45 |
| Pathology centre | 0.16 | ** | 0.06 | -0.35 |  | 0.25 |
| **Appointment type** |  |  |  |  |  |  |
| In-person | 0.12 |  | 0.09 | -0.12 |  | 0.26 |
| Phone call | -0.13 | ** | 0.06 | -0.03 |  | 0.37 |
| Video call | 0.07 |  | 0.06 | -0.10 |  | 0.25 |
| Online chat | -0.06 |  | 0.06 | 0.25 |  | 0.37 |
| **Extra services** |  |  |  |  |  |  |
| PrEP and STI-care | -0.08 | ** | 0.03 | 0.03 |  | 0.25 |
| PrEP, STI and general healthcare | 0.08 | ** | 0.03 | -0.03 |  | 0.25 |
| **Opt-out** | -2.36 | *** | 0.16 | 1.35 | ** | 0.68 |
| Log-likelihood function | -2088 |  |  |  |  |  |
| AIC/N | 1.676 |  |  |  |  |  |

* p value <0·10, ** p value <0·05, *** p value <0·01; ^#^ The coefficient for the reference group is calculated as the negative sum of the other coefficients.

Currency is in Australian dollars (1 AUD=0.64 USD).

AIC = Akaike information criteria; GP = General practitioner; HIV = Human immunodeficiency virus; LGBTQ+ = lesbian, gay, bisexual, trans and queer/questioning, and more identities; PrEP = pre-exposure prophylaxis (for HIV); SE = standard error; STI = sexually transmitted infections

# Supplementary Table S4 Latent class analysis of preferences of people without HIV and not using PrEP

|  | Just make it free  (22.8%) | | | General practice  (44.7%) | | | Don’t want to test  (22.2%) | | | Unsure about preferences  (10.2%) | | |
| --- | --- | --- | --- | --- | --- | --- | --- | --- | --- | --- | --- | --- |
|  | Coefficient |  | SE | Coefficient |  | SE | Coefficient |  | SE | Coefficient |  | SE |
| **Cost (AUD)** |  |  |  |  |  |  |  |  |  |  |  |  |
| $0 | 6.55 | *** | 1.46 | 0.14 | ** | 0.07 | 2.75 | *** | 0.20 | 0.85 | * | 0.46 |
| $50 | -0.16 |  | 0.40 | 0.25 | *** | 0.06 | -0.32 |  | 0.21 | -0.89 |  | 0.65 |
| $100 | -2.35 | *** | 0.56 | -0.13 | ** | 0.06 | -0.73 | *** | 0.19 | -0.66 |  | 0.57 |
| $150 | -4.04 | *** | 0.67 | -0.26 | *** | 0.07 | -1.70 | *** | 0.30 | 0.70 |  | 0.68 |
| **Where samples are taken** |  |  |  |  |  |  |  |  |  |  |  |  |
| Staff takes in clinic | 0.99 | * | 0.56 | -0.05 |  | 0.10 | -0.21 |  | 0.29 | 1.05 |  | 0.64 |
| Self-sampling in clinic | 0.64 |  | 0.52 | -0.03 |  | 0.10 | 0.21 |  | 0.25 | 1.14 | ** | 0.57 |
| Self-sampling at home | -0.77 | * | 0.42 | -0.09 |  | 0.07 | -0.02 |  | 0.20 | -1.28 | * | 0.69 |
| Pathology centre | -0.86 | * | 0.48 | 0.17 | ** | 0.07 | 0.02 |  | 0.19 | -0.91 | * | 0.54 |
| **Appointment type** |  |  |  |  |  |  |  |  |  |  |  |  |
| In-person | 0.68 | * | 0.35 | 0.18 | ** | 0.07 | 0.18 |  | 0.18 | 9.30 |  | 2514 |
| Phone call | 0.47 |  | 0.36 | 0.01 |  | 0.07 | 0.16 |  | 0.20 | 9.90 |  | 2506 |
| Video call | -0.52 |  | 0.39 | -0.26 | *** | 0.07 | -0.26 |  | 0.19 | -10.94 |  | 7071 |
| Online chat | -0.63 | * | 0.34 | 0.07 |  | 0.07 | -0.08 |  | 0.19 | -8.26 |  | 3576 |
| **Type of clinic** |  |  |  |  |  |  |  |  |  |  |  |  |
| Sexual health clinic | 0.54 |  | 0.35 | -0.08 |  | 0.06 | 0.20 |  | 0.17 | -13.58 |  | 2709 |
| GP with expertise in LGBTQ+ health | -0.36 |  | 0.33 | 0.35 | *** | 0.06 | -0.06 |  | 0.19 | 5.26 |  | 903 |
| Mainstream GP clinic | -0.49 |  | 0.32 | -0.06 |  | 0.06 | -0.33 | * | 0.19 | 5.91 |  | 903 |
| Peer-run testing service | 0.31 |  | 0.35 | -0.21 | *** | 0.06 | 0.19 |  | 0.17 | 2.41 |  | 903 |
| **Extra services** |  |  |  |  |  |  |  |  |  |  |  |  |
| HIV test only | -0.31 |  | 0.21 | -0.17 | *** | 0.05 | -0.19 |  | 0.16 | 0.20 |  | 0.45 |
| HIV and STI test/management | 0.44 |  | 0.29 | 0.10 | ** | 0.05 | 0.25 | * | 0.14 | 1.16 | ** | 0.48 |
| HIV and STI care and general healthcare | -0.13 |  | 0.24 | 0.07 |  | 0.05 | -0.06 |  | 0.14 | -1.36 | ** | 0.64 |
| **Appointment frequency** |  |  |  |  |  |  |  |  |  |  |  |  |
| 3 monthly | -0.62 | ** | 0.29 | 0.13 | ** | 0.05 | -0.02 |  | 0.14 | 0.70 |  | 0.46 |
| 6 monthly | 0.32 |  | 0.26 | -0.06 |  | 0.05 | 0.18 |  | 0.15 | 0.07 |  | 0.43 |
| Yearly | 0.30 |  | 0.25 | -0.07 |  | 0.05 | -0.16 |  | 0.14 | -0.77 |  | 0.56 |
| **Opt-out** | -5.19 | *** | 0.91 | -2.70 | *** | 0.23 | 1.29 | *** | 0.20 | 17.38 |  | 2663 |
| Log-likelihood function | -2424 |  |  |  |  |  |  |  |  |  |  |  |
| AIC/N | 1.503 |  |  |  |  |  |  |  |  |  |  |  |

* p value <0·10, ** p value <0·05, *** p value <0·01; ^#^ The coefficient for the reference group is calculated as the negative sum of the other coefficients. Currency is in Australian dollars (1 AUD=0.64 USD). AIC = Akaike information criteria; GP = General practitioner; HIV = Human immunodeficiency virus; LGBTQ+ = lesbian, gay, bisexual, trans and queer/questioning, and more identities; PrEP = pre-exposure prophylaxis (for HIV); SE = standard error; STI = sexually transmitted infections

# Supplementary Figure S1 Relative importance of preferences for people living with HIV

# Supplementary Figures S2 Relative importance of preferences for PrEP users

#

# Supplementary Figure S3 Relative importance of preferences of GBM without HIV and not using PrEP
